# Representation of Multiple Cellular Phenotypes Within Tissue-Level Simulations of Cardiac Electrophysiology

**DOI:** 10.1007/s11538-018-0516-1

**Published:** 2018-10-05

**Authors:** Louise A. Bowler, David J. Gavaghan, Gary R. Mirams, Jonathan P. Whiteley

**Affiliations:** 1 Department of Computer Science, University of Oxford, Oxford, UK; 2 Centre for Mathematical Medicine and Biology, School of Mathematical Sciences, University of Nottingham, Nottingham, UK

**Keywords:** Homogenisation, Monodomain, Bidomain, Cardiac electrophysiology, Stem cell-derived cardiomyocytes

## Abstract

Distinct electrophysiological phenotypes are exhibited by biological cells that have differentiated into particular cell types. The usual approach when simulating the cardiac electrophysiology of tissue that includes different cell types is to model the different cell types as occupying spatially distinct yet coupled regions. Instead, we model the electrophysiology of well-mixed cells by using homogenisation to derive an extension to the commonly used monodomain or bidomain equations. These new equations permit spatial variations in the distribution of the different subtypes of cells and will reduce the computational demands of solving the governing equations. We validate the homogenisation computationally, and then use the new model to explain some experimental observations from stem cell-derived cardiomyocyte monolayers.

## Introduction

Since its inception in the 1960s, the field of computational cardiac electrophysiology has contributed to many advances in understanding the links between the flow of ions, transmembrane potential and electromechanical activity of the heart under control, pathological and drug-influenced conditions. In particular, much attention has been devoted to modelling the *action potential* within cardiac tissue—that is, the transmembrane potential at a given location, as a function of time, during a given cardiac cycle—and the extracellular potential, allowing simulation of electrocardiograms. Mathematical models are now available of the action potentials observed in many different species and cardiac cell types (Noble and Rudy 2001; Fink et al. 2011).

In this paper, we develop methods for simulating a system that is of particular interest for safety pharmacology—monolayers of human stem cell-derived cardiomyocytes (hSC-CMs). The Comprehensive in vitro Proarrhythmia Assay (CiPA) initiative has proposed a series of complementary cardiac safety assays to improve upon the current methods of assessing the arrhythmic risk associated with novel pharmaceutical compounds (Sager et al. 2014; Gintant et al. 2016). The use of hSC-CMs will form a key component of the CiPA paradigm through multi-cellular assays such as the micro-electrode array (Harris et al. 2013; Clements and Thomas 2014).

**Fig. 1:**
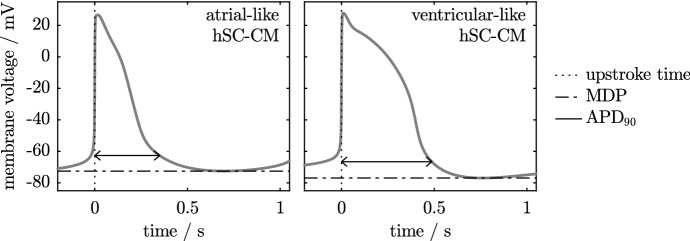
Simulated action potentials of atrial-like and ventricular-like human stem cell-derived cardiomyocytes, generated using the Paci et al. (2013) model. Two properties of the action potentials are indicated. The maximum diastolic potential, MDP, is the most hyperpolarised potential. The action potential duration, $\hbox {APD}_{90}$, is the time taken to achieve a given percentage (here, 90%) of repolarisation following the upstroke

The action potentials of hSC-CMs are often classified into one of the three subpopulations, or phenotypes, two of which are shown in Fig. 1. The consequences of having different phenotypes of cell within a small tissue sample are difficult to investigate experimentally. We therefore propose simulation as a method by which the impact of variation in cell type on the cardiac safety assessment process may be investigated. In this paper, we compare two methods of simulating a system that contains variable cellular populations, with our primary focus on a future application to simulation of multi-cellular hSC-CM cultures.

### Characteristics of Human Stem Cell-Derived Cardiomyocytes

Human stem cell-derived cardiomyocytes are electrophysiologically and structurally immature, with some of their properties resembling neonatal cells rather than their adult counterparts. They are small and rounded, with diameters of approximately 10–50 µm [see for example Snir et al. (2003), Gherghiceanu et al. (2011) or Fig. 1 in Ma et al. (2011)]. hSC-CMs typically beat spontaneously, with multi-cellular cultures exhibiting a focus of activation, or pacemaker region, which triggers excitation in the remainder of the culture. The activation wavefronts travel at slower speeds than observed in adult cardiac tissue, usually in the region of 2–20 cm/s (Burridge et al. 2011; Mehta et al. 2011; Lee et al. 2012).

As this system is made up of coupled oscillators, from a mathematical perspective we might expect to observe synchronous activation throughout the monolayer (Mirollo and Strogatz 1990). However, finite conduction velocities are observed.

Analysis of hSC-CM action potentials from single cells has indicated that three subpopulations, or phenotypes, may be present within a given sample of cells: atrial-like, ventricular-like and nodal-like (He et al. 2003; Zhang et al. 2009; Ma et al. 2011). The phenotypes are named to reflect the similarity with the action potentials found in the respective regions of the adult heart, and are usually defined in terms of metrics based on the duration of the action potential, although alternatives have also been proposed (Lopez-Redondo et al. 2016). Precise statistics on the relative abundance of each phenotype are difficult to obtain due to different methods of classification and inherent variability within each of the phenotypes (Pekkanen-Mattila et al. 2010). At the present time, there are differing views on the spatial organisation of these phenotypes within tissue. Zhu et al. (2016) and Vestergaard et al. (2017) reported regions of different action potential morphology within some, but not all, clusters of human embryonic stem cell-derived cardiomyocytes. However, Du et al. (2015) did not detect such spatial organisation in their studies of monolayers of human-induced pluripotent stem cell-derived cardiomyocytes, instead reporting a spectrum of action potential morphologies throughout the tissue.

### Existing Methods for Tissue Simulations Containing Multiple Electrophysiology Phenotypes

Tissue-level cardiac electrophysiology is usually modelled using the monodomain or bidomain equations (Keener and Sneyd 2009). When modelling multiple phenotypes, the tissue is usually partitioned into regions containing only one phenotype. However, this method becomes computationally infeasible if the phenotypes are well-mixed within the tissue, as the tissue must be partitioned into many very small regions where just a single phenotype is present. Under these conditions, we may utilise the extended bidomain (or tridomain) model. The extended bidomain model adds a second intracellular domain for a second phenotype and has been used to simulate mixtures of cardiomyocytes and fibroblasts (Sachse et al. 2009) and gastrointestinal electrophysiology (Buist and Poh 2010). The two intracellular domains represent continuously linked regions of each of the two types of cell; a third intracellular domain would be required if it were to be used for simulating the three cell types reported in hSC-CM cultures. The extended bidomain model is well-suited for simulating thoroughly mixed cell types (Corrias et al. 2012), with the two interconnected intracellular domains providing a natural method by which two cell types can be considered to occupy a small unit of space. To model spatial variation in phenotype proportions in the extended bidomain model, we could adjust the surface area of each domain per unit volume of tissue ($\chi$). But, as Sachse et al. (2009) observed, “it is unclear [how we should adjust the intra- and inter-domain gap junction conductivities to model] the density and arrangement of myocytes and fibroblasts”. These authors linearly scaled the conductivities in each domain from values in tissues with 100% myocytes or 100% fibroblasts according to volume fraction of each phenotype. Based on the assumption that within each domain there are always connections between cells of a given phenotype, the number of connections is proportional to the volume fraction. These assumptions may not hold in regions with low proportions of a phenotype: with only say 10% of a given cell type present, a typical cell of that type does not ‘touch’ and share gap junctions with any cells of the same type, there may be no continuous domain of this cell type through which currents can flow.

We therefore take an alternative and perhaps simpler approach and develop a modified derivation of the bidomain equations that assumes a mixture of cell types within the repeating homogenisation unit that is used in their derivation.

### Outline of Study

The overall goal of this study is to model the electrophysiological properties of cardiac tissue containing multiple cellular subpopulations by extending the derivation of the standard bidomain equations to permit the modelling of more than one cell type. The equations governing this model are derived in “Appendix A” and summarised in Sect. 2. In Sect. 3, we propose a suite of simulations, designed with two aims in mind: to verify the derivation of the model, and to illustrate some key properties of systems that contain more than one cellular population. We present the results of the simulations in Sect. 4. Finally, in Sect. 5, we conclude by discussing how these simulations can inform investigation of hSC-CM monolayers in a two-dimensional domain.

## The Mathematical Model

**Fig. 2:**
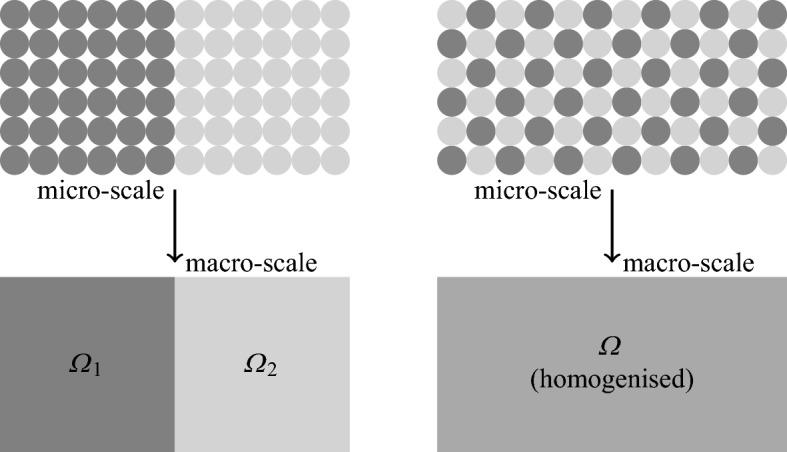
Different spatial distributions of cellular phenotype. The case on the left may be divided into two partitioned regions, each containing a single type of cell. Partitioning the case on the right into single-phenotype regions would result in many tiny partitions. Performing the homogenisation process over regions containing both types of cell is therefore preferable

As explained in Sect. 1.1, we require a mathematical model that includes more than one cellular phenotype. We consider two possibilities: tissue that may be partitioned into distinct regions that each contain only one cellular phenotype; and tissue where all phenotypes are well-mixed. These two situations are shown in Fig. 2. The derivation of this model (including all assumptions made) may be found in “Appendix A”, where the equations are written in nondimensional form. In dimensional form, these governing equations are

1 $$\begin{aligned} \chi \bigg (C_{m} \frac{\partial V}{\partial t} + I_{\text {ion}}\bigg )= & {} \nabla \cdot \left( \varSigma _{i} \nabla (V + \phi _{e}) \right) , \end{aligned}$$

2 $$\begin{aligned} 0= & {} \nabla \cdot \left( \varSigma _{i} \nabla V + \left( \varSigma _{i} + \varSigma _{e} \right) \nabla \phi _{e} \right) , \end{aligned}$$

where *V* is the transmembrane potential, $\phi _{e}$ is the extracellular potential, $\chi$ is the ratio of cell membrane area to volume, $C_{m}$ is the capacitance of the membrane, $I_{\text {ion}}$ is the ionic current per unit area (specified by the solution of a system of ordinary differential equations at each point in space), and $\varSigma _{i},\varSigma _{e}$ are the intracellular and extracellular conductivity tensors. The surface area of the cell mebmrane consists of a fraction $\rho _{1}$ of phenotype 1, and a fraction $\rho _{2}=1-\rho _{1}$ of phenotype 2. If the capacitance of these phenotypes are $C_{m1},C_{m2}$, and the ionic current densities per unit area are $I_{\text {ion},1},I_{\text {ion},2}$ we have

3 $$\begin{aligned} C_{m}= & {} \rho _{1}\, C_{m1} + \rho _{2}\, C_{m2}, \end{aligned}$$

4 $$\begin{aligned} I_{\text {ion}}= & {} \rho _{1}\, I_{\text {ion},1} + \rho _{2}\, I_{\text {ion},2}. \end{aligned}$$

When modelling hSC-CMs, both the intracellular and extracellular conductivity tensors may be approximated as isotropic. As a consequence $\varSigma _i = \alpha \varSigma _e$, and the bidomain equations simplify to the monodomain equation (Keener and Sneyd 2009):

5 $$\begin{aligned} \chi \left( C_m \frac{\partial V}{\partial t} + I_\text {ion}(V; \mathbf {u})\right) - \frac{\partial }{\partial x} \left( \varSigma \frac{\partial V}{\partial x}\right) = 0, \end{aligned}$$

where

$$\begin{aligned} \varSigma = \varSigma _i \left( \varSigma _i + \varSigma _e\right) ^{-1} \varSigma _e = \frac{\alpha }{1+\alpha } \varSigma _e. \end{aligned}$$

When the cells are not well-mixed, we may instead partition the tissue region into regions where only one phenotype is present and solve the monodomain equations on each of these regions; we refer to this model as the partitioned phenotypes (PP) model.

## Description of Simulations

Our initial simulations validate the governing equations by comparing several properties of the action potential, for both the PP and HP models, in the limit that the separation of scales parameter [defined in Eq. (22)] $\delta \rightarrow 0$. We then demonstrate that the HP model may be used to reproduce experimental observations. Our simulations are all of a single fibre in one spatial dimension. Our boundary conditions enforce no flow of current across either end of the fibre and may be written

6 $$\begin{aligned} \frac{\partial V}{\partial x} = 0,\qquad \text {at both ends of the fibre.} \end{aligned}$$

This fibre is made up of two different cell types. In the absence of experimental evidence indicating any variation in conductivities, we assume constant intra- and extracellular conductivities throughout the fibre.

### Simulation Sets

The simulations may be divided into six sets. In the first five sets, we use the phenomenological FitzHugh–Nagumo (FHN) action potential model (FitzHugh 1961; Nagumo et al. 1962) where

7 $$\begin{aligned} I_{\text {ion}}(V;w)= & {} V(V-\alpha )(1-V)-w, \end{aligned}$$

8 $$\begin{aligned} \frac{\mathrm{d}w}{\mathrm{d}t}= & {} \epsilon V - \beta w. \end{aligned}$$

This model is described by only three parameters, $\alpha , \beta , \epsilon$, which are allowed to vary spatially to take account of different phenotypes. In particular, the model is self-exciting (i.e. does not require a stimulus to excite) if $\alpha < 0$, and excitable (i.e. requires a sufficiently large external stimulus to excite) if $\alpha \ge 0$. The linearity of the model in $\alpha , \beta$ and $\epsilon$ allows the parameters for the HP model to be calculated very easily using Eq. (4). For example, a parameter $\alpha$ appears in the FHN model in Eq. (7). In the HP model, we denote this parameter by $\alpha _{H}$, and see that it takes the value

9 $$\begin{aligned} \alpha _H = \rho _1\alpha _1 + \rho _2\alpha _2 . \end{aligned}$$

The values of $\beta _{H}$ and $\epsilon _H$ (the values of $\beta$ and $\epsilon$ used in the HP model) may be calculated in a similar manner. As such, the FHN model allows us to compare the predicted excitability properties of the HP model very effectively. In the final set of simulations, we use the physiological Paci et al. (2013) models of atrial-like and ventricular-like hSC-CM electrophysiology to investigate our intended use-case of simulating a monolayer of hSC-CMs, as used in safety pharmacology assays.

**Fig. 3:**
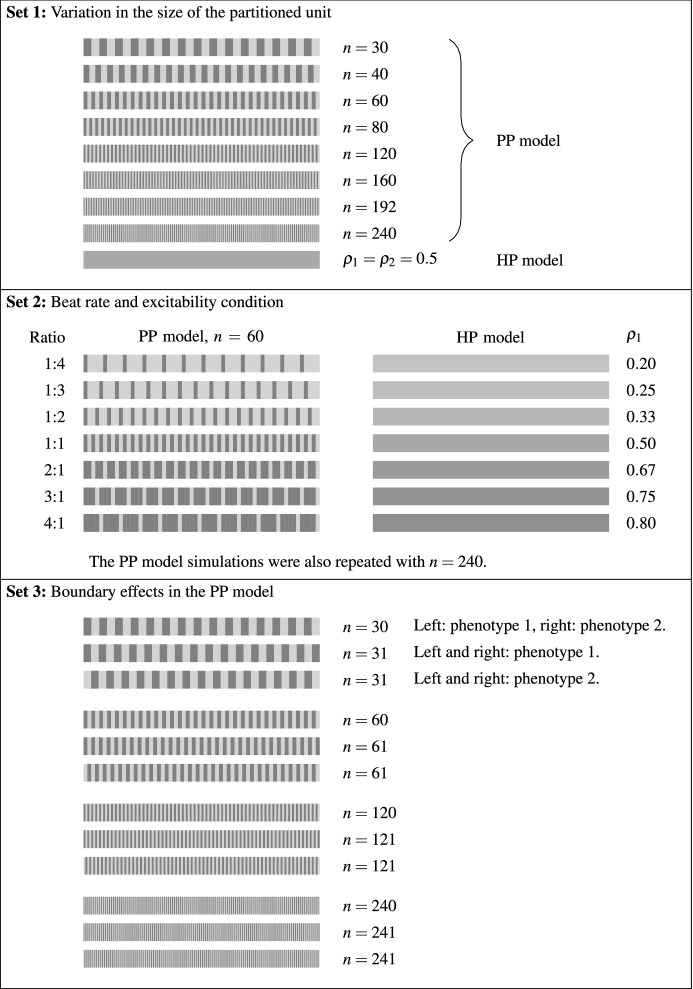
Spatial layout of the different phenotypes in the first three sets of simulations. The dark and light shades represent regions within which one of two cellular phenotypes is exclusively present. The cellular electrophysiology models that represent the two cell types are chosen from the six parameterisations of the FitzHugh–Nagumo model described in the main text. Intermediate shades denote the HP model with appropriate values of $\rho _1$ and $\rho _2$ (the relative contributions of each phenotype). The value of *n* indicates the number of regions into which the domain was partitioned when the PP model was used

*Set 1* This set of simulations is designed to test whether the action potentials of the PP model tend towards those of the HP model as the size of the unit that we homogenise over is decreased; that is, in the limit $\delta \rightarrow 0$, where $\delta$ is defined in “Appendix A”. This is achieved by varying *n*, the number of repeating units that the domain is divided into. The layout of phenotypes is shown in Fig. 3. Four different combinations of parameterisations of the FitzHugh–Nagumo model are investigated, with each pair having different combinations of positive or negative $\alpha$ values. The simulations are run until the action potential on each cardiac cycle is identical to that on the previous cycle.

*Set 2* In a further stage of verification of the homogenisation, we use a small size of partitioned unit and alter the relative proportions of the two model phenotypes, $\rho _1$ and $\rho _2$, throughout the series of simulations, and compare the beat rates from both models. We see in Eq. (9) that varying $\rho _1$ and $\rho _2$ alters the excitability properties of the HP model, and so this set of simulations allows us to verify that the excitability properties of the HP model are correctly predicted. The layout of phenotypes is shown in Fig. 3.

*Set 3* We will see later that the first two sets of simulations exhibit some localised behaviour in the vicinity of boundaries. Our third set of simulations investigates these effects through varying the distribution of phenotypes close to the boundary, as shown in Fig. 3.

**Fig. 4:**
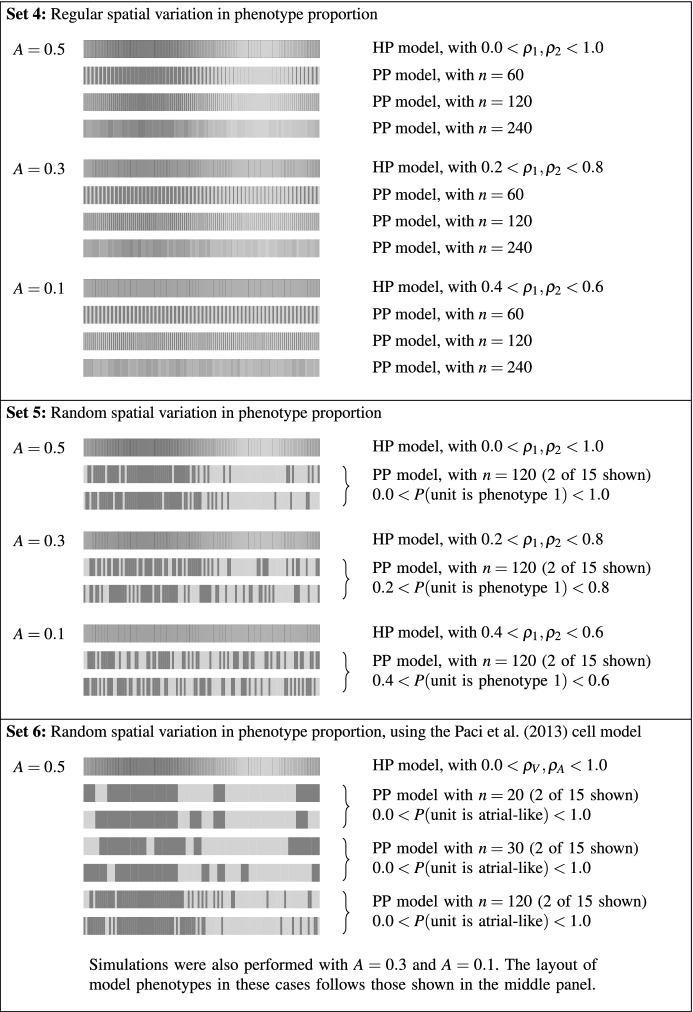
Spatial layout of the different phenotypes in the final three sets of simulations. As in the previous figures, the dark and light shades represent two different phenotypes. In Sets 4 and 5, we utilise the parameterisations of the FitzHugh–Nagumo model that are listed in the main text, while in Set 6 we use atrial-like and ventricular-like models of hSC-CM electrophysiology (Paci et al. 2013)

*Set 4* Until now, our investigations have assumed constant proportions of the two model phenotypes throughout the fibre at the macroscale. This assumption is, however, likely to be unrealistic. We therefore consider a fibre where the values of $\rho _1$ and $\rho _2$ used in the HP model are given by

10 $$\begin{aligned} \rho _1(x)&= 0.5 + A\sin \left( \frac{2\pi x}{L}\right) , \end{aligned}$$

11 $$\begin{aligned} \rho _2(x)&= 1 - \rho _1(x), \end{aligned}$$

where *L* is the length of the fibre, and *A* is the amount of variation in $\rho _1$ and $\rho _2$, with $A\le 0.5$ so that $0 \le \rho _1,\rho _2 \le 1$. In the PP model, the fibre is first divided into *n* units. In each unit, both $\rho _1$ and $\rho _2$ are calculated at the mid-point of the unit using Eqs. (10) and (11). The first fraction $\rho _1$ of the unit is designated as phenotype 1; the remainder is designated as phenotype 2, as illustrated in Fig. 4.

*Set 5* In these simulations, the values of $\rho _1$ and $\rho _2$ used in the HP model are identical to those described in Set 4. We introduce some random variation into the values of $\rho _1$ and $\rho _2$ used in the PP model. We first generate *n* uniformly distributed random numbers, $R_{1},R_{2},\ldots ,R_{n}$, between 0 and 1, where *n* is the number of partitioned units. Let $x_{m}$ be the *x*-coordinate of partitioned unit *m*. We then assign partitioned unit *m* as phenotype 1 if

12 $$\begin{aligned} R_{m} \le 0.5+A\sin \left( \frac{2\pi x_{m}}{L}\right) , \end{aligned}$$

and phenotype 2 otherwise. We also impose the restriction that an equal number of units with each of the two phenotypes are present in the fibre by rejecting any phenotype layout if this restriction is not met. For each of our choices of parameter *A*, we simulate 15 different arrangements of model phenotypes. Some example distributions of phenotypes are given in Fig. 4.

*Set 6* Our final set of simulations closely follows the design of those in Set 5, but a physiological cell model is used rather than a phenomenological model. The first cell model is the ventricular-like model of Paci et al. (2013), while the second model is the atrial-like model from the same paper. In addition to altering the parameter *A*, we also alter the number of units, *n*, that the fibre is partitioned into. Example phenotype distributions can be found in Fig. 4.

### Parameters Used in the Simulations

**Fig. 5:**
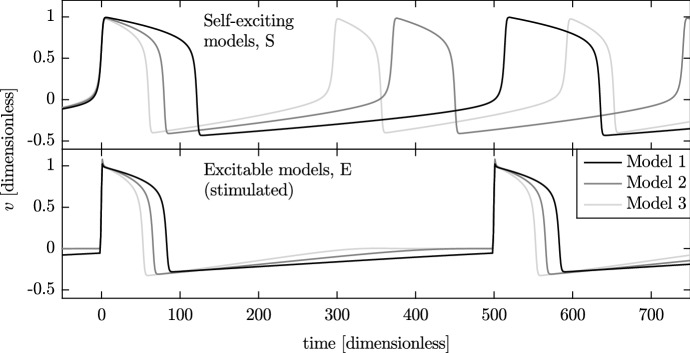
Action potentials of the six parameterisations of the FitzHugh–Nagumo model. The three self-exciting models (top) beat at their natural frequencies, while the excitable models (bottom) are stimulated every 500 time units. Upstroke times have been aligned at $\text {time} = 0$

We define six sets of the $\alpha$, $\beta$ and $\epsilon$ parameters for three self-exciting (S1–S3) and three excitable (E1–E3) models. The parameters chosen for these models result in action potentials with clearly different beat rates and action potential durations and have a range of positive and negative $\alpha$ values. The parameters of the six forms of the FHN model are listed in Table 1, producing the action potentials shown in Fig. 5.

**Table 1:** Values of the FitzHugh–Nagumo model parameters $\alpha , \beta$ and $\epsilon$

Name	$\alpha$	$\beta$	$\epsilon$	Rate	$\hbox {APD}_{90}$	MDP
Model S1	$-$ 0.12	$2\times 10^{-7}$	0.002	0.0019	122	$-$ 0.433
Model S2	$-$ 0.08	$3\times 10^{-7}$	0.003	0.0027	81.3	$-$ 0.410
Model S3	$-$ 0.06	$4\times 10^{-7}$	0.004	0.0034	61.6	$-$ 0.400
Model E1	0.12	$2\times 10^{-7}$	0.002	N/A	84.6	$-$ 0.281
Model E2	0.08	$3\times 10^{-7}$	0.003	N/A	67.1	$-$ 0.310
Model E3	0.06	$4\times 10^{-7}$	0.004	N/A	54.3	$-$ 0.326

These sets of parameters were used to produce the different action potentials as shown in Figure 5. Alongside, we list the dimensionless values of spontaneous beat rate, action potential duration ($\hbox {APD}_{90}$) and maximum diastolic potential (MDP) for each model

In our final set of simulations, we use the Paci et al. (2013) physiologically based cellular electrophysiology model, with other parameters given in the right-hand column of Table 2.

**Table 2:** Tissue-level parameters for monodomain simulations

Parameter	Value (FHN simulations)	Value (Paci et al. (2013) simulations)
$C_m$	1	$1\,\upmu \hbox {F}\,\hbox {cm}^{-2}$
$\chi$	1	$1400\,\hbox {cm}^{-1}$
$\varSigma$	1	$0.3\, \hbox {mS}\,\hbox {cm}^{-1}$
*x*-domain	$0{-}100$	$0{-}1\,\hbox {cm}$
*x*-step	0.013	0.00052 cm
Simulation duration	8000	20 s
Time step (PDE)	$2^{-10}$	$2.5\times 10^{-4}\,\hbox {s}$
Time step (ODE)	$2^{-10}$	$5\times 10^{-6}\,\hbox {s}$
Initial conditions	$v=1\times 10^{-3}$	As listed in the supplement of
	$w=0$	Paci et al. (2013)
Stimulus period	500	N/A
Stimulus duration	2	N/A
Stimulus magnitude	$-\,0.4$ at $0<x<x_\text {end}/30$	N/A

The stimulus was only applied when the model combinations present in the fibre would not otherwise spontaneously activate. All quantities in the FHN simulations are dimensionless. Values of *x*-step were chosen so that at least 32 or 16 [FHN and Paci et al. (2013) simulations, respectively] finite elements were present in the smallest size of partitioned unit

The monodomain problem was solved numerically using a custom implementation of the piecewise linear finite element method in Matlab. The systems of ordinary differential equations from either the FitzHugh–Nagumo or Paci et al. (2013) models were solved using the Forward Euler method. Accuracy of the solver was checked by comparing output against the analytical solution of an example one-dimensional monodomain problem from Pathmanathan and Gray (2014). Convergence studies were performed on systems based on the first set of simulations. The selected values of the space and time-steps are listed in Table 2, along with other relevant simulation parameters.

## Results of Simulations

We now perform the simulations described in Sect. 3.

### Set 1: Variation in the Size of the Partitioned Unit

As we described in Sect. 3.1, the primary aim of these simulations is to validate our homogenisation procedure. We do this by examining two cell-level properties of the action potential, $\hbox {APD}_{90}$ and maximum diastolic potential, and one tissue-level property, conduction velocity. We use the layout of phenotypes shown in the top panel of Fig. 3 with four different combinations of the cellular electrophysiology models described in Sect. 3.2, given by:

- *Models S1 and S3* Both self-exciting, with $\alpha _H=-0.09$;
- *Models S1 and E2* Self-exciting and excitable, respectively, with $\alpha _H=-0.02$;
- *Models S3 and E2* Also self-exciting and excitable, but with $\alpha _H=0.02$; and
- *Models E1 and E3* Both excitable, with $\alpha _H=0.09$.

Using $\rho _{1}=\rho _{2}=0.5$ in these initial simulations, we therefore expect the first two combinations to be self-exciting in the PP model in the limit that the number of compartments *n* increases; for the second two combinations, a stimulus will be required. A stimulus at the left-hand end of the fibre was used for combinations that were not self-exciting.

#### $\hbox {APD}_{90}$ and Maximum Diastolic Potential

**Fig. 6:**
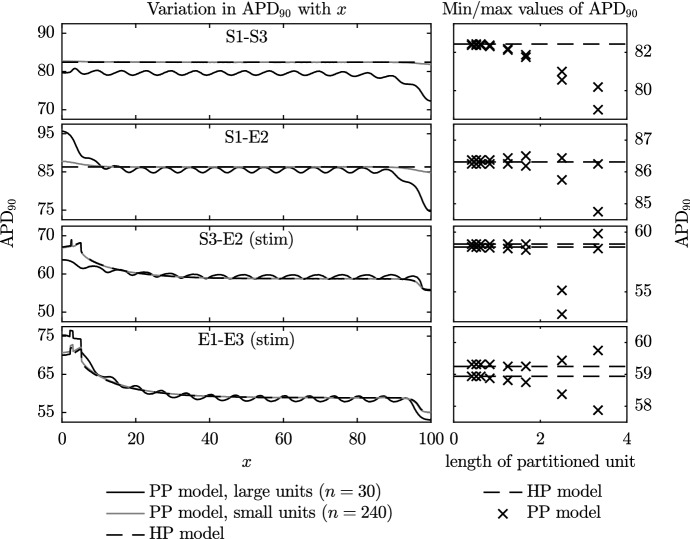
Variation in $\hbox {APD}_{90}$ (time to achieve $90\%$ repolarisation) during the final beat in Set 1 simulations. The panels on the left show the variation in $\hbox {APD}_{90}$ across the fibre during the final complete beat for three selected cases: those of the PP model with the largest and smallest partitioned regions, and the HP model. The panels on the right show the minimum and maximum values of $\hbox {APD}_{90}$ across the central region of $35<x<65$ during the final complete beat of all Set 1 simulations. Values from the PP model are shown using crosses, while those from the HP model are indicated with the dotted line

**Fig. 7:**
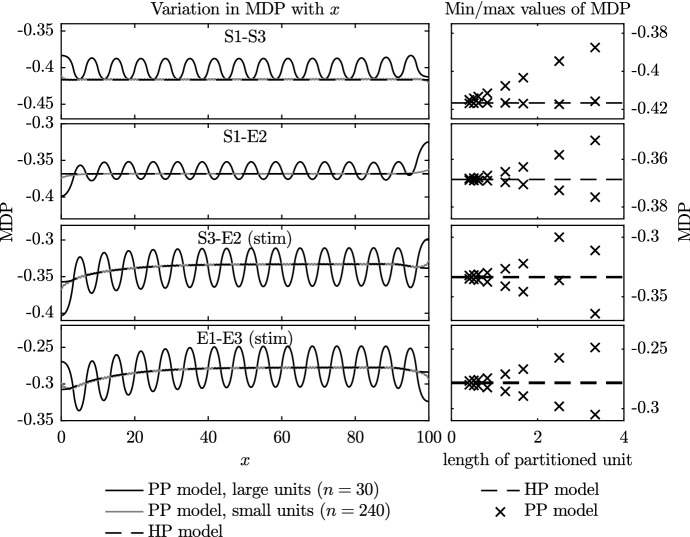
Variation in maximum diastolic potential (MDP) from Set 1 simulations. As in the previous figure, the panels on the left show the MDP across the entire fibre for selected cases. Panels on the right provide a summary of the minimum and maximum values of MDP recorded in all simulations

In Fig. 6, for each of the four different combinations of model phenotype, we plot the action potential duration for the PP simulations with varying numbers of partitioned units, *n*, and the HP simulations. On the left of the figure, we plot the $\hbox {APD}_{90}$ along the entire fibre for: the HP model; the PP model with small partitioned units ($n=240$); and the PP model with large partitioned units ($n=30$). On the right-hand side, we plot the minimum and maximum values of the $\hbox {APD}_{90}$ that occur over the central region of the fibre as *n* varies. As the stimulus can have a substantial effect on the value of the $\hbox {APD}_{90}$ and other properties of the action potential, only the values between $x=35$ and $x=65$ are taken into account in these plots. In Fig. 7, we plot the maximum diastolic potential (MDP) in a similar manner to the $\hbox {APD}_{90}$ values that are plotted in Fig. 6.

The results shown in Figs. 6 and 7 generally follow a smooth trend in that, as the size of the partitioned unit decreases, the $\hbox {APD}_{90}$ and MDP of the PP model approach those of the HP model. This confirms that the homogenisation process has worked as expected.

In Figs. 6 and 7, we note that the $\hbox {APD}_{90}$ and MDP vary across the fibre. This variation becomes more marked near to the boundaries. This is because a travelling wave action potential, i.e. $V=f(x-ct)$ (where *c* may depend on all variables and parameters in the model) is unable to satisfy the boundary condition given by Eq. (6), as has previously been noted by Cherry and Fenton (2011) in a single-phenotype study. We investigate this phenomenon in more detail in Sect. 4.3, but first make some comments that may be explained using these initial simulations.

There are two reasons for the boundary effects that can be observed in plots of $\hbox {APD}_{90}$ across the domain in Fig. 6 where we have spatially alternating phenotype partitions. Initially we consider regions distant from any boundaries. If a phenotype A has a longer single-cell $\hbox {APD}_{90}$ than a phenotype B then, upon repolarisation in the PP tissue simulation, current flows from more depolarised to less depolarised regions, which means that phenotype B’s repolarisation is delayed by both its neighbouring A phenotypes. The same currents cause phenotype A’s repolarisation to be encouraged by both its neighbouring B phenotypes, and the overall effect is to smooth the $\hbox {APD}_{90}$ along the fibre. However, a phenotype on the boundary has just one neighbouring phenotype partition, with a no-flux boundary condition at the other side, which means that these smoothing effects are reduced and its $\hbox {APD}_{90}$ phenotype can become more dominant. The difference between the single-cell $\hbox {APD}_{90}$ values of the phenotypes themselves ($\hbox {APD}_{90}$ shown in Table 1) then dictates the magnitude of this effect (S1–E2 have a large $\hbox {APD}_{90}$ difference of approximately 55 ms, and large edge effects; whereas S3–E2 have a difference of only 5.5 ms and much smaller edge effects).

We also see edge effects due to wave propagation: an action potential reaching a boundary exhibits a shortened $\hbox {APD}_{90}$ due to the no-flux condition instead of the presence of a more depolarised wave ahead; and conversely prolonged $\hbox {APD}_{90}$ when an action potential originates on a boundary (as studied in detail by Cherry and Fenton (2011)). This second effect occurs in a homogenous phenotype situation as well, and so we deduce it is the dominant cause of the boundary effects in the lower two cases of Fig. 6 as both the HP and PP models exhibit similar edge effects.

As the MDP is a property of the action potential during the hyperpolarised or resting phase, the influence of the pacemaker location on its value is smaller than for the $\hbox {APD}_{90}$. As we observed for the $\hbox {APD}_{90}$, the nature of the boundary conditions pulls the MDP higher or lower than would otherwise be expected at the boundary. The one exception is again related to the pacemaking site, with the minimum value of MDP being slightly higher than expected at the right-hand side of the S1–S3 fibre.

There are, however, two exceptions to the smooth trends that we now explain. The first exception is in the two lower plots on the right-hand side of Fig. 6. We note that the $\hbox {APD}_{90}$ seen across the region $35< x < 65$ does still exhibit variations, albeit small variations, as the size of the partitioned unit decreases. This is because both of these simulations are excitable, rather than self-exciting, and therefore require a stimulus (artefact of stimulus edge can be seen on far left). Since the subsequent behaviour is asymmetric the influence of the stimulus prolongation can still be seen in the $35< x < 65$ domain.

The second exception is in the third panel down on the right of Fig. 6, where we see an outlying result. For a large partitioned unit, where we may not expect the homogenisation to be valid, this PP model was self-exciting despite $\alpha _H$ being positive. The region of the self-exciting Model S3 closest to the boundary was able to spontaneously depolarise as it was separated from the influence of non-self-exciting Model E2 by the entire large length of the partitioned unit. As a stimulus was also applied, the action potentials switched between spontaneous and stimulated. The change in effective beat rate has an impact on the $\hbox {APD}_{90}$, which can be seen in Fig. 6: the final beat in the simulation with the second-largest partitioned unit was spontaneous rather than triggered by the stimulus.

#### Conduction Velocity

**Fig. 8:**
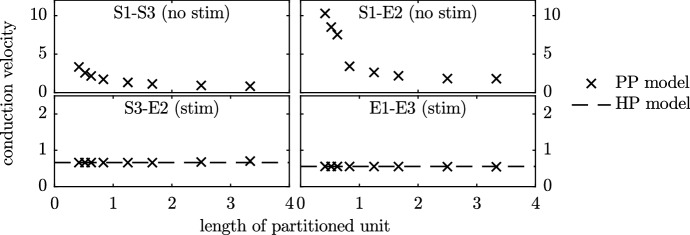
Conduction velocity of the travelling waves in the Set 1 simulations. Activation times in cases where the HP model was used to simulate a spontaneously activating system (i.e. models S1–S3 and S1–E2 in the top panels) were synchronous, leading to an infinite conduction velocity

In Fig. 8, we plot the conduction velocity of the HP and PP models when the four phenotype combinations were used. The conduction velocity was calculated through the region $35<x<65$. When the HP model was used for the self-exciting systems, full synchronisation occurred and the activation time was identical throughout the entire fibre, and so conduction velocity is evaluated as infinite. Consequently, the top two panels do not contain lines for the conduction velocity of the HP model. As the length of the partitions in the PP model case decreases towards zero, we expect to tend towards the synchronised HP case (where each point in space has each model present). Indeed, we see the effective conduction velocity increase to high above the stimulated case, as synchronisation effects occur and begin to dominate. We note that the magnitude of the increase in conduction velocity observed in these simulated systems is far greater than that typically reported as a result of restitution effects (Yue et al. 2005; Zhang et al. 2012).

In the non-self-exciting stimulated fibres (the lower panels of Fig. 8), we observe good agreement between the conduction velocity for the HP and PP models for all lengths of partitioned unit.

### Set 2: The Beat Rate and Excitability Condition When Phenotype Proportions Vary

In our second stage of the verification of the homogenisation, we investigate whether the beating condition of the FitzHugh–Nagumo model ($\alpha <0$ for spontaneous beating) holds, and compare the beat rates of fibres simulated using the PP and HP models.

We pair each self-activating action potential model (S1, S2, S3) with each of the excitable models (E1, E2, E3). We alter the relative proportions of the two models, $\rho _1$ and $\rho _2$, in both the HP and PP fibres, giving us a range of model combinations with different values of the homogenised parameter $\alpha _H=\rho _1\alpha _1+\rho _2\alpha _2$. We perform two sets of simulations with the PP model, one with mid-sized partitioned regions ($n=60$) and another with much smaller partitioned regions ($n=240$). The partitioned units are assigned a model phenotype in a regular pattern. Rather than alternating the phenotype of each partitioned unit, the precise layout is determined by the relative proportions of the two phenotypes (see Fig. 3 for further details).

**Fig. 9:**
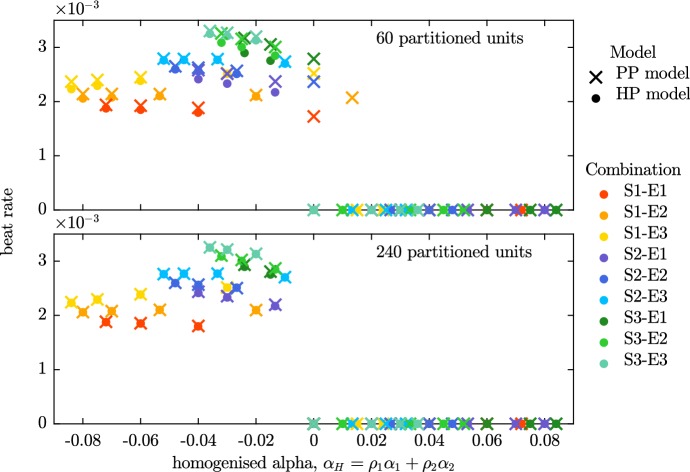
Beat rate from Set 2 simulations. The beat rates of fibres simulated using the PP model are compared to those of fibres simulated using the HP model with equivalent proportions of the two phenotypes. With both sizes of partitioned units, the discrepancies between the HP and PP models (indicated by the proximity of the cross-dot pairs) are generally small. The discrepancies are noticeably smaller in the lower panel, where the smaller partitioned units are used. The major difference between the small and large partitioned unit simulations may be seen around $\alpha _H=0$. The HP model is quiescent at this value of $\alpha _H$, as are all instances of the PP model with small partitioned units. However, spontaneous beating is still seen in some of the simulations that utilise the PP model with larger partitioned units (Color Figure Online)

We first examine the discrepancy in beat rate between the HP and PP models in both panels of Fig. 9. The beat rates of the HP and PP models differ more when the partitioned units are large (top panel) than when the partitioned units are small (bottom panel). In general the condition that the PP model is self-exciting only when $\alpha _H>0$ is adhered to. The only places where this condition is not met is around $\alpha _H=0$, for the case where $n=60$. This problem disappears when $n=240$, i.e. when the size of the partitioned unit decreases and $\delta$ approaches zero, and we are closer to the limit in which our homogenisation is valid.

### Set 3: Boundary Effects

In Sect. 4.1.1, we noted the presence of boundary effects in both the PP and HP models. To examine this effect, we alter the model phenotype that is located at the boundary of three otherwise similar phenotype layouts for the PP model. We use Models S1 and S3 for this investigation as their spontaneous activity is representative of the beating of hSC-CMs. In the first case, where we have an even number of partitioned units, the outer model phenotype on the left-hand side of the fibre is Model S1 (with a slower natural frequency), while that at the right is Model S3 (with a faster natural frequency). In the other two cases, we have an odd number of units (one more than in the even-*n* cases). In one of these, Model S1 is present at both boundary units, while in the other Model S3 is present at both boundaries. We test these three patterns of model phenotypes with partitioned units in a range of sizes.

**Fig. 10:**
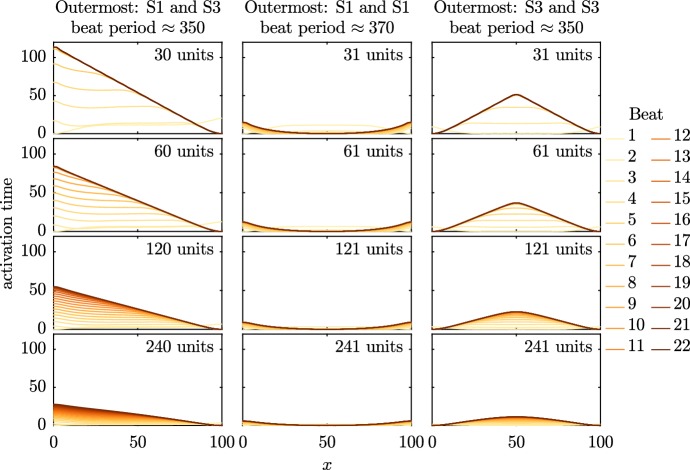
Activation times of all recorded beats in PP model simulations (Set 3) with equally sized partitions. The activation times are normalised so that the earliest activation time during each beat is set to 0. Model S1 has a slower natural beat rate than Model S3, and so propagation spreads from regions where Model S3 can dominate. As the number of partitions increases, the apparent conduction velocity increases due to synchronisation effects as we tend to the homogenised case (Color Figure Online)

We plot the activation times of every beat in each of the three cases in Fig. 10. In each row of the figure, we move to a smaller size of the partitioned unit: at the top, we have 30 or 31 units, with 60 or 61, 120 or 121, and 240 or 241 in the rows below. In all cases plotted in Fig. 10, several beats occur before activation settles into a steady pattern. As we start off with identical initial conditions across the entire fibre, the first beat occurs near-simultaneously across the entire fibre before the dominant pacemaker begins to take over. While the process of the pacemaker settling to a steady state is induced by the initial conditions in this case, similar effects have been reported in experimental systems following other forms of perturbation (Kienast et al. 2014).

Throughout the simulations shown in Fig. 10, the origin of activation is consistent as the number of partitioned units is increased. The activation wave always originates from a region of the faster-beating model phenotype; if Model S3 is present at one of the boundaries, the activation wave originates there. In the central case, activation begins at the central instance of Model S3, as the slower-beating Model S1 takes longer to reach the activation threshold at the boundaries than it does elsewhere. The increase in conduction velocity as the size of the partitioned unit decreases shows that the action potentials tend towards synchronisation in the homogenised limit, as we saw in Fig. 8.

### Set 4: Regular Spatial Variation in Phenotype Proportion

**Fig. 11:**
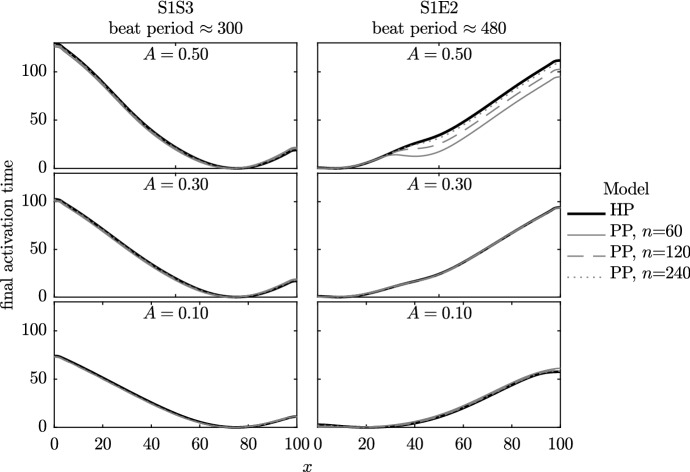
Activation time of the final beat for Set 4 simulations. We compare the homogenised phenotypes (HP) model (black line) to the partitioned phenotypes (PP) model with 60, 120 and 240 partitioned units. Parameter *A* controls the extent of variation in phenotype—with more variation in phenotype there is a slower wave speed in both HP and PP models and a more noticeable difference between the HP and large-unit PP models. See Fig. 4 for the underlying phenotype arrangements across this domain

In Fig. 11, we plot the final activation time from a series of simulations where $\rho _1$ and $\rho _2$ are allowed to vary spatially. We alter the parameter *A* from Eqs. (10)–(12) across our three investigations, to give us three distributions of the two model phenotypes with different amounts of variability across the fibre (see Fig. 4 for details). This series of simulations shows that as the size of the partitioned unit is decreased, the PP model tends towards the HP model.

### Set 5: Random Spatial Variation in Phenotype Proportion

**Fig. 12:**
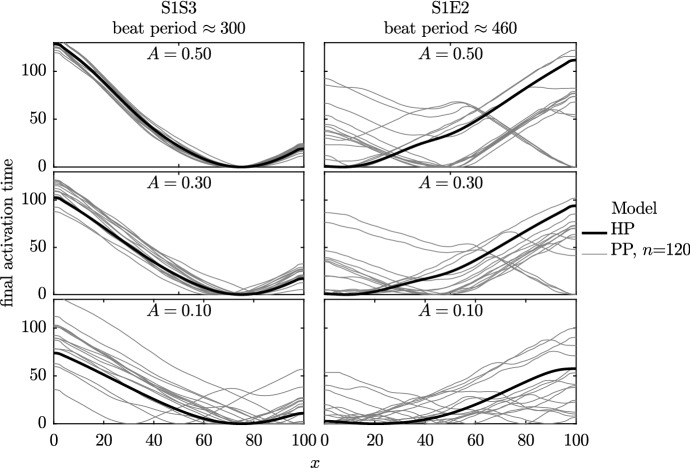
Activation times of the final beat in the Set 5 simulations. The bold black line shows the activation time from the homogenised phenotypes model. The thinner grey lines show the activation time from the 15 randomly assigned partitions for the PP model [probabilities given by Eq. (12)], so that each has a slightly different phenotype layout, examples of which may be seen in Fig. 4. The HP model and PP model wave speeds are in good agreement (the same gradients are seen in these activation time plots), but the random arrangement of phenotype partitions can change the location of the emergent ‘pacemaker’ site(s) in the PP model

In Sect. 4.4, we investigated a smooth variation in $\rho _1$ and $\rho _2$. We now add a random perturbation onto this smooth distribution, as described in Sect. 3.1. In Fig. 12, we plot the activation time of the final beat in as a bold black line for the homogenised case and grey thin lines for the partitioned cases. We have 15 repeats of the PP model simulations due to the different random arrangements of model phenotypes, as shown in Fig. 4, where $n=120$.

In contrast to the simulations for a regular variation in $\rho _1$ and $\rho _2$ shown in Fig. 11, we see that introducing randomness into $\rho _1$ and $\rho _2$ can induce differences between the PP and HP models. Specifically, the location of the pacemaker region may differ between these models, particularly in the simulations where the smooth variation is small (i.e. *A* is small), as the variations due to random error are then relatively large when compared to *A*. We note, however, that the gradients of all activation plots are very similar. Hence, although the pacemaker region may not be accurately located using the HP model, the conduction velocity is consistent. We return to this point when considering a physiological cell model in Sect. 4.6.

### Set 6: Simulations with Physiological Action Potential Models

In this set of simulations, we extend the simulations in Sect. 4.5 to use the Paci et al. (2013) models of atrial-like and ventricular-like hSC-CM electrophysiology. The atrial-like model has a faster beat rate and shorter $\hbox {APD}_{90}$ than the ventricular-like model, as is shown previously in Fig. 1. We arrange these two phenotypes in a similar manner to that used in the previous series of simulations, which can be seen in the second panel of Fig. 4. We investigate the impact of varying the parameter *A*, which sets the amount of variation in phenotype across the fibre.

**Fig. 13:**
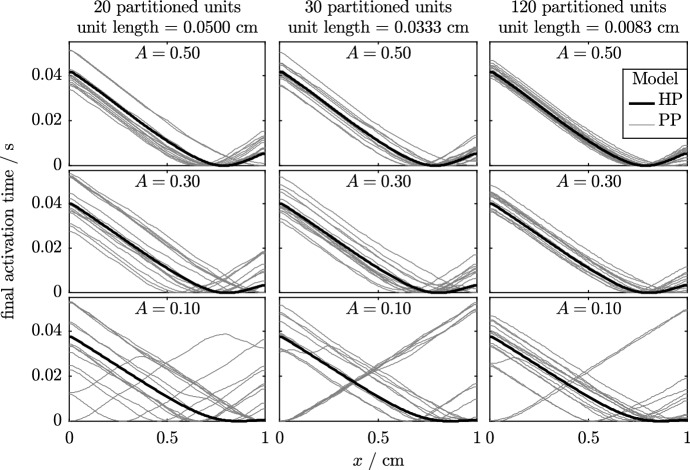
Activation times of the final beat of simulations in the Set 6 simulations. The two cell types are represented by the Paci et al. (2013) models of ventricular-like and atrial-like hSC-CM electrophysiology. Results from the homogenised phenotypes model are shown as a bold black line; the thin grey lines represent the 15 randomly generated partitions according to Eq. (12). Each repeat has a slightly different phenotype layout; see Fig. 4 for examples of the underlying phenotype arrangements across this domain. As we noted in the previous set of simulations, the HP model and PP model wave speeds are in good agreement and we observe that the random arrangement of phenotype partitions can change the emergent ‘pacemaker site’ in the PP model

In Fig. 13, we plot the final activation times of the simulations with varied numbers of partitioned units and values of *A*. From left to right, the figure shows the activation times of fibres divided into 20, 30 and 120 partitioned units. As we are now dealing with a dimensional simulation, this corresponds to patches of cells that are approximately 500, 330 and 80 µm across. The typical size of a hSC-CM varies, but even the smallest size of partitioned unit that is tested here would correspond to at least two cells. As in the previous section, we decrease the amount of variability in phenotype from top to bottom of the figure, with parameter *A* set to 0.5, 0.3 and 0.1 in each row.

In the previous section, we noted that the HP model is able to capture the overall behaviour of the PP model very well when there is substantial variation in phenotype, i.e. a high value of *A*. We make similar observations to those made in Fig. 13—as *A* is progressively increased, keeping *n* fixed, we see that the pacemaker region is accurately located by the HP model. Even if the pacemaker region is not accurately located, it is seen that the conduction velocity is accurately predicted, as can be seen by the gradient of the plot of activation times. We also observe that the pacemaker region is more accurately located as *n* increases, as expected. Finally, we note from the gradient of the activation plots that the conduction velocity is around $19\,\text {cm/s}$ for all values of *A*, which is similar to that discussed in Sect. 1.1.

## Conclusions

We have investigated two models for including multiple cellular phenotypes within simulations of cardiac tissue. In the partitioned phenotypes (PP) model, the simulated domain contains distinct regions where a single-model phenotype is present. The homogenised phenotypes (HP) model assumes a well-mixed sample of cells, which we represent as a homogenised system. We have verified that the electrical activity generated by the PP model tends towards that of the HP model as the size of the partitions decreases. The HP model is therefore a good approximation to the PP model when the length scale of regions containing a mixture of cell types is small.

Use of the PP model requires that the mesh is sufficiently fine in order to capture the geometry of the partitioned regions as closely as possible. For realistic two- and three-dimensional simulations with small regions of distinct cell types, this will result in a very large number of nodes, and simulations using this mesh may not be computationally feasible. An advantage of the HP model is that it does not require the mesh to explicitly model the geometry of the partitioned regions, thus significantly reducing the number of nodes in the mesh and eliminating the need for customised versions of the mesh when simulating the same domain with different arrangements of cell types.

Our simulations have demonstrated some experimentally observed properties of hSC-CM monolayers. The first two sets of simulations involved fibres with regularly repeating units of alternating phenotype, tending towards a fully mixed system. We observed that changes in values of $\hbox {APD}_{90}$ and MDP were apparent across the fibre, with the changes being gradual despite clear division between cell types in the PP model. The conduction velocity of the activation wave increased rapidly when a self-activating cell model was present in fibres simulated with the small-unit PP model or the HP model. More realistic conduction velocities were seen in simulations where there was spatial variation in the distribution of phenotypes. The lack of a dramatic variation in conduction velocity in experimental hSC-CM systems, such as those described in Lee et al. (2012), suggests that spatially homogeneous cellular phenotypes are unlikely to occur in cultures of hSC-CMs; and that there must be variation in the intrinsic beat rate of hSC-CMs in these multi-cellular cultures. This prediction is consistent with two other recent modelling studies: Abbate et al. (2018) and Tixier et al. (2018) propose that there must be spatial variation in phenotype in hSC-CMs to provoke signals of the magnitude observed in micro-electrode array experiments. In these papers, different phenotypes were introduced with a partitioned phenotype, and with a smoothly varying parameter set within one model, respectively.

The final three sets of simulations demonstrated how local spatial variability in the relative proportions of the two phenotypes introduced a stable pacemaker region in the HP and small-unit PP models. This observation provides a mechanism by which stable propagation of the activation wave can occur in hSC-CMs, even in cultures that only exhibit small amounts of variation in phenotype. The sixth set of simulations utilised physiologically based models of atrial-like and ventricular-like hSC-CM electrophysiology. We demonstrated similar conduction velocities in cases where both large and small amounts of phenotypic variation were simulated, in the region of values observed experimentally, which vary from approximately 1 cm/s to 20 cm/s depending on maturity (Mehta et al. 2011; Lee et al. 2012; Zhu et al. 2017). We can therefore propose that even a small amount of phenotypic variation removes the system from the fully synchronous regime observed when the HP model was used in the first set of simulations. However, synchronisation may still play a small role in the value of conduction velocity: our observations lead us to the prediction that paced hSC-CM monolayers may show slower conduction velocities than they do when left to self-excite.

In future work, we will compare two-dimensional simulation results using this model with experimental measurements from approximately two-dimensional monolayers of stem cell-derived cardiomyocytes. Such experiments typically use micro-electrode arrays to record extracellular potential at a number of sites in the centre of a monolayer in a circular well, and so provide some information on the direction and speed of propagating waves.

In addition to our main focus of human stem cell-derived cardiomyocytes, the homogenised phenotypes model may also be useful in other types of cardiac simulation where two or more cell types are present, such as in sino-atrial node where cellular properties are reported to vary based on their position within the pacemaking region. The current interest in uncertainty quantification and variability in biological systems is driven by the need to understand how these factors can affect model output, thus influencing the utility of these models to complement experiments (Elkins et al. 2013; Mirams et al. 2016). Our proposals for simulation of multiple cell types will enable detailed investigation of the impact of variable spatial distributions of cell type on the signals recorded from monolayer cultures of human stem cell-derived cardiomyocytes that are part of the proposed Comprehensive in vitro Proarrhythmia Assay initiative (Sager et al. 2014).

## Figures and Tables

**Fig. 14:**
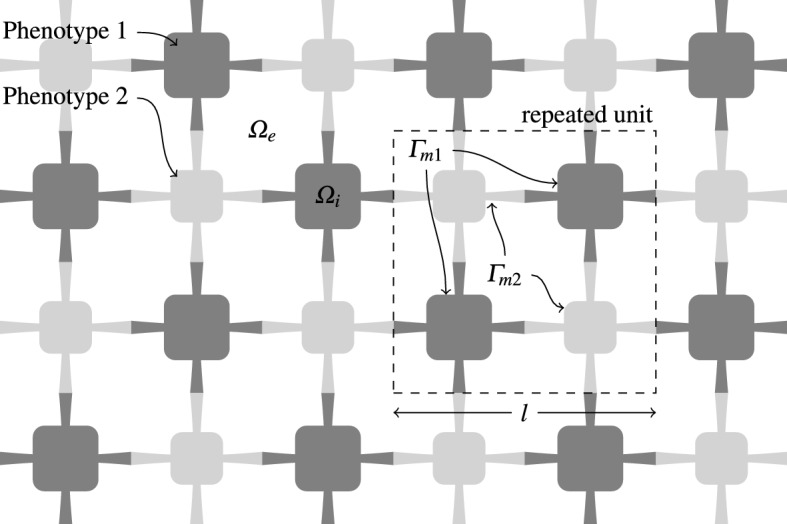
Cells of two different phenotypes in the HP model. The repeated unit is made up of both cell types; it should be noted that the two types do not have to be present in the same proportions. $\varOmega _i$ and $\varOmega _e$ denote the intracellular and extracellular domains, respectively. The surface area of the cell membrane within the repeated unit is given by $\varGamma _m = \varGamma _{m1} + \varGamma _{m2}$. The lengthscale of the solution, *L*, is assumed to be much larger than the lengthscale of the repeated unit, *l*

## Derivation of the Mathematical Model

The derivation of the HP model is very similar to the derivation of the bidomain equations given by other authors (Neu and Krassowska 1993; Keener and Panfilov 1996; Keener and Sneyd 2008; Hand and Griffith 2010, 2011; Richardson and Chapman 2011; Bruce et al. 2014). By including different cell types in the spatial unit that we homogenise over, we must take account of cell parameters that depend on the microscale coordinate. We therefore follow the approach of Bruce et al. (2014) who allowed some cell parameters to vary when modelling gap junctions. The derivation of the PP model is simpler than that of the HP model and follows naturally from the derivation of the latter. We therefore describe the derivation of the HP model first, and then explain how this may be modified to obtain the PP model.

In Sect. A.1, we begin by writing down a discrete model that partitions cardiac tissue into an intracellular region and an extracellular region, and nondimensionalise the governing equations in Sect. A.2. We then set the scene for the homogenisation by defining the macroscale and microscale coordinates, and the periodic unit that we homogenise over, in Sect. A.3.1. We proceed with the derivation of the homogenised equations in intra- and extracellular space in Sects. A.3.2 and A.3.3 allowing us to write down the HP model for well-mixed cellular phenotypes in Sect. A.4. The collapse of the HP model to the PP model is described in Sect. A.5.

### The Discrete Domains Model

We assume that cardiac tissue occupying a region can be partitioned into an intracellular region denoted by $\varOmega _{i}$, and an extracellular region denoted by $\varOmega _{e}$. These two regions are separated by the cell membrane, assumed to be of negligible thickness, denoted by $\varGamma _{m}$. The intracellular space and extracellular space have scalar conductivities $\sigma _{i}$ and $\sigma _{e}$, respectively. We will allow $\sigma _{i}$ to vary spatially to take account of different cell types, but will assume that $\sigma _{e}$ is constant. By Ohm’s law, the intracellular and extracellular currents are given by

$$\begin{aligned} {\mathbf {i}}_{i}&=-\sigma _{i} \nabla \phi _{i}, \qquad {\mathbf {x}}\in \varOmega _{i}, \\ {\mathbf {i}}_{e}&=-\sigma _{e} \nabla \phi _{e}, \qquad {\mathbf {x}} \in \varOmega _{e}, \end{aligned}$$

where $\phi _{i},\phi _{e}$ are the intracellular and extracellular potentials. Conservation of current in the intracellular and extracellular space then gives

13 $$\begin{aligned} \nabla \cdot \left( \sigma _{i} \nabla \phi _{i} \right)&=0, \qquad {\mathbf {x}} \in \varOmega _{i}, \end{aligned}$$

14 $$\begin{aligned} \nabla \cdot \left( \sigma _{e} \nabla \phi _{e} \right)&=0, \qquad {\mathbf {x}} \in \varOmega _{e}. \end{aligned}$$

The boundary conditions that model the flux of current across the cell membrane may be written

15 $$\begin{aligned} -\sigma _{i} \nabla \phi _{i} \cdot {\mathbf {n}} = I_{m}, \qquad -\sigma _{e} \nabla \phi _{e} \cdot {\mathbf {n}} = -I_{m}, \qquad {\mathbf {x}} \in \varGamma _{m}, \end{aligned}$$

where ${\mathbf {n}}$ is the unit vector, normal to $\varGamma _{m}$, that points from the intracellular space into the extracellular space, and $I_{m}$ is the transmembrane current per unit area flowing into the intracellular space from the extracellular space. Modelling the cell membrane as a capacitor, $I_{m}$ is given by

16 $$\begin{aligned} I_{m} = C_{m} \frac{\partial v}{\partial t} + I_{\text {ion}}, \end{aligned}$$

where $C_{m}$ is the capacitance of the membrane per unit area, $v=\phi _{i}-\phi _{e}$ is the transmembrane potential, *t* is time, $I_{\text {ion}}(v;{\mathbf {u}})$ is the net current per unit area due to the flux of ions across the membrane, and ${\mathbf {u}}$ contains quantities that are specified by a model of the flow of ions across the cell membrane. As we are modelling more than one cell type we allow both $C_{m}$ and $I_{\text {ion}}$ to vary spatially.

### Nondimensionalisation

We nondimensionalise the equations given in Sect. A.1 using the following scalings:

$$\begin{aligned} t&= T {\hat{t}}, \qquad \mathbf {x}= L {\hat{\mathbf {x}}}, \qquad \sigma _{i} = {\bar{\varSigma }} {\hat{\sigma }}_{i}, \qquad \sigma _{e} = {\bar{\varSigma }} {\hat{\sigma }}_{e}, \\ \phi _{i}&= \varPhi {\hat{\phi }}_{i}, \qquad \phi _{e} = \varPhi {\hat{\phi }}_{e}, \qquad v = \varPhi {\hat{v}}, \qquad C_{m} = {\bar{C}} {\hat{C}}_{m} \qquad I_{\text {ion}} = {\bar{I}} {\hat{I}}_{\text {ion}}, \end{aligned}$$

where *T* is a typical timescale, *L* is a typical lengthscale for the solution (rather than the length of a myocyte), ${\bar{\varSigma }}$ is representative of the scalar conductivities, $\varPhi$ is representative of the potential difference across the cell membrane, ${\bar{C}}$ is representative of the capacitance, and ${\bar{I}}$ is representative of the ionic current per unit area. Equations (13) and (14) may then be written in nondimensional form as

17 $$\begin{aligned} \nabla \cdot \left( {\hat{\sigma }}_{i} \nabla {\hat{\phi }}_{i} \right)&=0, \qquad \hat{{\mathbf {x}}} \in \varOmega _{i}, \end{aligned}$$

18 $$\begin{aligned} \nabla \cdot \left( {\hat{\sigma }}_{e} \nabla {\hat{\phi }}_{e} \right)&=0, \qquad \hat{{\mathbf {x}}} \in \varOmega _{e}, \end{aligned}$$

and we can reformulate the boundary conditions of Eq. (15) using the definition of $I_m$ from Eq. (16) to give

19 $$\begin{aligned} -{\hat{\sigma }}_{i} \nabla {\hat{\phi }}_{i} \cdot \hat{{\mathbf {n}}}&= {{\mathcal {A}}} {\hat{C}}_{m} \frac{\partial {\hat{v}}}{\partial {\hat{t}}} + {{\mathcal {B}}} {\hat{I}}_{\text {ion}}, \qquad \hat{{\mathbf {x}}} \in \varGamma _{m}, \end{aligned}$$

20 $$\begin{aligned} -{\hat{\sigma }}_{e} \nabla {\hat{\phi }}_{e} \cdot \hat{{\mathbf {n}}}&= -{{\mathcal {A}}} {\hat{C}}_{m} \frac{\partial {\hat{v}}}{\partial {\hat{t}}} - {{\mathcal {B}}} {\hat{I}}_{\text {ion}}, \qquad \hat{{\mathbf {x}}} \in \varGamma _{m}, \end{aligned}$$

where the nondimensional constants ${{\mathcal {A}}}$ and ${{\mathcal {B}}}$ are given by

21 $$\begin{aligned} {{\mathcal {A}}} = \frac{{\bar{C}} L}{T {\bar{\varSigma }}}, \qquad {{\mathcal {B}}} = \frac{{\bar{I}} L}{\varPhi {\bar{\varSigma }}}. \end{aligned}$$

For the remainder of Sect. A, we use the nondimensional equations (17)–(20) presented above, dropping hats for clarity.

### Derivation of the Homogenised Equations

To allow us to homogenise the equations presented in Sect. A.2, we make the assumption that cardiac tissue is a periodic lattice of repeating cuboid units, where each unit contains a small number of cardiac cells. We show a representation of this lattice in two dimensions in Fig. 14. No assumptions are made regarding the type of cells within each unit of the lattice: we allow the cells to have different shapes, sizes, capacitances and conductivities, and also allow the ionic current passing through the cell membrane to take different functional forms depending on which cell type the membrane belongs to.

#### The Domains and Coordinate Systems

We assume that the lengthscale for the solution, *L*, is much greater than the lengthscale of each unit in the lattice, *l*. We then define the nondimensional parameter $\delta$ by

22 $$\begin{aligned} \delta = \frac{\text {lengthscale of unit we homogenise over (=l)}}{\text {lengthscale for solution (=L)}}, \end{aligned}$$

and note that our assumptions on the lengthscales imply that $\delta \ll 1$.

Before deriving the bidomain equations, we first verify that our assumption that $\delta \ll 1$ is valid. In Sect. 1.1, we explained that hSC-CMs are small and rounded, with diameters of approximately 10–50 µm. Setting $l=200$ µm allows us to homogenise over a unit containing several cells. Further, $L=2000$ µm is a representative lengthscale of the solution, yielding $\delta =0.1$, and $T=10^{-2}$ s is a representative timescale of the solution. Other parameters that appear in the nondimensional constants ${{\mathcal {A}}}$ and ${{\mathcal {B}}}$ defined by Eq. (21) are: ${\bar{C}}=1$ µF $\hbox {cm}^{-2}$ (from Table 2); ${\bar{\varSigma }} = 0.3$  mS $\hbox {cm}^{-1}$ (from Table 2); ${\bar{I}}=2\times 10^{-5}$ A $\hbox {cm}^{-2}$ [from Paci et al. (2013)]; and $\varPhi =7 \times 10^{-2}$ V [from (Paci et al. 2013)]. We may then deduce that the nondimensional constants ${{\mathcal {A}}}$ and ${{\mathcal {B}}}$ defined by Eq. (21) satisfy ${{\mathcal {A}}} = {{\mathcal {O}}}(\delta )$ and ${{\mathcal {B}}} = {{\mathcal {O}}}(\delta )$, and write

23 $$\begin{aligned} {{\mathcal {A}}} = {{\mathcal {A}}}_{1}\delta , \qquad {{\mathcal {B}}} = {{\mathcal {B}}}_{1}\delta , \end{aligned}$$

where ${{\mathcal {A}}}_{1} = {{\mathcal {O}}}(1)$, and ${{\mathcal {B}}}_{1} = {{\mathcal {O}}}(1)$. This is identical to the distinguished limit investigated by Richardson and Chapman (2011) when deriving the tissue scale bidomain equations and is consistent with the parameters used in typical bidomain simulations; see, for example, Morgan et al. (2009) and Bishop and Plank (2012).

We will utilise the separation of scales described above by introducing a microscale coordinate $\mathbf {z}$, defined by

24 $$\begin{aligned} \mathbf {z}=\frac{1}{\delta }\mathbf {x}, \end{aligned}$$

where $\mathbf {x}$ is the macroscale coordinate used in Sect. A.1.

#### Derivation of the Homogenised Equation in Intracellular Space

We write $\phi _{i}=\phi _{i}(\mathbf {x},\mathbf {z},t)$, where $\phi _{i}$ is periodic in $\mathbf {z}$. Using the definition of the microscale and macroscale coordinates given by Eq. (24), we see that

25 $$\begin{aligned} \nabla \phi _{i} = \nabla _{\mathbf {x}} \phi _{i} + \frac{1}{\delta } \nabla _{\mathbf {z}} \phi _{i}, \end{aligned}$$

where $\nabla _{\mathbf {x}}$ and $\nabla _{\mathbf {z}}$ are the gradient operators with respect to the $\mathbf {x}$ and $\mathbf {z}$ coordinates, respectively. We then write $\phi _{i}$ as a regular asymptotic expansion in the parameter $\delta$:

26 $$\begin{aligned} \phi _{i}(\mathbf {x},\mathbf {z},t) = \phi _{0}^{\text {(in)}}(\mathbf {x},\mathbf {z},t) + \delta \phi _{1}^{\text {(in)}}(\mathbf {x},\mathbf {z},t) + \delta ^{2} \phi _{2}^{\text {(in)}}(\mathbf {x},\mathbf {z},t) +\cdots , \end{aligned}$$

where all functions in the expansion are periodic in $\mathbf {z}$. We assume that $\sigma _{i} = \sigma _{i}(\mathbf {z})$ to allow for different conductivities should cell types with different conductivities be present in the unit that we homogenise over. Substituting Eqs. (25) and (26) into Eq. (17) and collecting equal powers of $\delta$ gives, for $\mathbf {z}\in \varOmega _{i}$.  Similarly using Eq. (19) and using Eq. (23), we may equate powers of $\delta$ to generate the following boundary conditions:

The differential equation Eq. (27), and boundary condition (30), are satisfied by

33 $$\begin{aligned} \phi _{0}^{\text {(in)}} = \phi _{0}^{\text {(in)}}(\mathbf {x},t), \end{aligned}$$

and so there is no $\mathbf {z}$ dependence in the solution for $\phi _{i}$ at leading order, as expected. We now turn our attention to the differential equation Eq. (28), and boundary condition (31). Equation (33) allows us to deduce that the first term on the left-hand side of Eq. (28) is zero. The structure of this equation and boundary condition suggests seeking a solution

34 $$\begin{aligned} \phi _{1}^{\text {(in)}}(\mathbf {x},\mathbf {z},t) = W_{1}^{\text {(in)}}(\mathbf {z}) \frac{\partial \phi _{0}^{\text {(in)}}}{\partial x_{1}} + W_{2}^{\text {(in)}}(\mathbf {z}) \frac{\partial \phi _{0}^{\text {(in)}}}{\partial x_{2}} + W_{3}^{\text {(in)}}(\mathbf {z}) \frac{\partial \phi _{0}^{\text {(in)}}}{\partial x_{3}}, \end{aligned}$$

for functions $W_{1}^{\text {(in)}},W_{2}^{\text {(in)}},W_{3}^{\text {(in)}}$ to be determined. The differential equation and boundary condition given by Eqs. (28) and (31) are then satisfied providing, for $j=1,2,3$,

35 $$\begin{aligned} \nabla _{\mathbf {z}} \cdot \left( \sigma _{i}(\mathbf {z}) \nabla _{\mathbf {z}} W_{j}^{\text {(in)}} \right)&= - \frac{\partial \sigma _{i}}{\partial z_{j}}, \qquad \mathbf {z}\in \varOmega _{i}, \end{aligned}$$

36 $$\begin{aligned} \left( \sigma _{i}(\mathbf {z}) \nabla _{\mathbf {z}} W_{j}^{\text {(in)}} \right) \cdot \mathbf {n}&= - \sigma _{i} n_{j}, \qquad \mathbf {z}\in \varGamma _{m}, \end{aligned}$$

where $W_{1}^{\text {(in)}},W_{2}^{\text {(in)}},W_{3}^{\text {(in)}}$ are periodic in $\mathbf {z}$, and $n_{j}$ is component *j* of $\mathbf {n}$, $j=1,2,3$.

Integrating Eq. (29), the differential equation at order $\delta ^{0}$, over the intracellular space component of the unit we are homogenising over (denoted by ${\hat{\varOmega }}_{i}$) yields

$$\begin{aligned} \int _{{\hat{\varOmega }}_{i}} \nabla _{\mathbf {x}} \cdot \left[ \sigma _{i}(\mathbf {z}) \left( \nabla _{\mathbf {x}} \phi _{0}^{\text {(in)}} + \nabla _{\mathbf {z}} \phi _{1}^{\text {(in)}} \right) \right] ~\mathrm{d}V_{\mathbf {z}} + \int _{{\hat{\varOmega }}_{i}} \nabla _{\mathbf {z}} \cdot \left[ \sigma _{i}(\mathbf {z}) \left( \nabla _{\mathbf {x}} \phi _{1}^{\text {(in)}} + \nabla _{\mathbf {z}} \phi _{2}^{\text {(in)}} \right) \right] ~\mathrm{d}V_{\mathbf {z}} = 0. \end{aligned}$$

Applying the divergence theorem to the second integral in the equation above gives

$$\begin{aligned} \int _{{\hat{\varOmega }}_{i}} \nabla _{\mathbf {x}} \cdot \left[ \sigma _{i}(\mathbf {z}) \left( \nabla _{\mathbf {x}} \phi _{0}^{\text {(in)}} + \nabla _{\mathbf {z}} \phi _{1}^{\text {(in)}} \right) \right] ~\mathrm{d}V_{\mathbf {z}} + \int _{\partial {\hat{\varOmega }}_{i}} \left[ \sigma _{i}(\mathbf {z}) \left( \nabla _{\mathbf {x}} \phi _{1}^{\text {(in)}} + \nabla _{\mathbf {z}} \phi _{2}^{\text {(in)}} \right) \right] \cdot \mathbf {n}~\mathrm{d}V_{\mathbf {z}} = 0. \end{aligned}$$

Using the boundary condition given by Eq. (32) and periodicity in $\mathbf {z}$ then gives

37 $$\begin{aligned} \int _{{\hat{\varOmega }}_{i}} \nabla _{\mathbf {x}} \cdot \left[ \sigma _{i}(\mathbf {z}) \left( \nabla _{\mathbf {x}} \phi _{0}^{\text {(in)}} + \nabla _{\mathbf {z}} \phi _{1}^{\text {(in)}} \right) \right] ~\mathrm{d}V_{\mathbf {z}} = \int _{\varGamma _{m}} {{\mathcal {A}}}_{1} C_{m} \frac{\partial v}{\partial t} + {{\mathcal {B}}}_{1} I_{\text {ion}}~\mathrm{d}S_{\mathbf {z}}. \end{aligned}$$

Denoting the volume of the repeating unit by $| \varOmega |$, Eqs. (33) and (34) then allow us to write

38 $$\begin{aligned} \nabla _{\mathbf {x}} \cdot \left( \varSigma _{i} \nabla _{\mathbf {x}} \phi _{0}^{\text {(in)}} \right)= & {} \frac{1}{| \varOmega |} \int _{\varGamma _{m}} \left( {{\mathcal {A}}}_{1} C_{m} \frac{\partial v}{\partial t} + {{\mathcal {B}}}_{1} I_{\text {ion}}\right) ~\mathrm{d}S_{\mathbf {z}}, \end{aligned}$$

39 $$\begin{aligned} \text {with}~\varSigma _{i}= & {} \frac{1}{| \varOmega |} \int _{{\hat{\varOmega }}_{i}} \sigma _{i}(\mathbf {z}) \left( \varvec{\mathcal {I}} + \frac{\partial {\mathbf {W}}^{\text {(in)}}}{\partial \mathbf {z}} \right) ~{\mathrm{d}}V_{\mathbf {z}}, \end{aligned}$$

where $\varvec{\mathcal {I}}$ is the identity matrix, and the matrix $\partial {\mathbf {W}}^{\text {(in)}}/\partial \mathbf {z}$ has entries given by

$$\begin{aligned} \left( \frac{\partial {\mathbf {W}}^{\text {(in)}}}{\partial \mathbf {z}} \right) _{jk} = \frac{\partial W_{j}^{\text {(in)}}}{\partial z_{k}}, \quad j,k=1,2,3. \end{aligned}$$

We note that the homogenised conductivity tensor would not be calculated in practice using Eq. (39) as this would require extremely high-resolution imaging in three-dimensional space to determine the domain occupied by a cell. Rather, Eq. (38) serves to demonstrate the structure of the differential equation satisfied by the leading order intracellular potential. The entries of the homogenised conductivity tensor are determined by fitting to experimental values of conduction velocity.

#### The Homogenised Equation in Extracellular Space

Using a similar argument, we may also write $\phi _{e}$ as a regular asymptotic expansion in the variable $\delta$:

$$\begin{aligned} \phi _{e}(\mathbf {x},\mathbf {z},t) = \phi _{0}^{\text {(ex)}}(\mathbf {x},\mathbf {z},t) + \delta \phi _{1}^{\text {(ex)}}(\mathbf {x},\mathbf {z},t) + \delta ^{2} \phi _{2}^{\text {(ex)}}(\mathbf {x},\mathbf {z},t) +\cdots , \end{aligned}$$

where all functions in the expansion are periodic in $\mathbf {z}$, and deduce that

40 $$\begin{aligned} \nabla _{\mathbf {x}} \cdot \left( \varSigma _{e} \nabla _{\mathbf {x}} \phi _{0}^{\text {(ex)}} \right)= & {} -\frac{1}{| \varOmega |} \int _{\varGamma _{m}} \left( {{\mathcal {A}}}_{1} C_{m} \frac{\partial v}{\partial t} + {{\mathcal {B}}}_{1} I_{\text {ion}}\right) ~\mathrm{d}S_{\mathbf {z}}, \end{aligned}$$

41 $$\begin{aligned} \text {where}~\varSigma _{e}= & {} \frac{1}{| \varOmega |} \int _{{\hat{\varOmega }}_{e}} \sigma _{e} \left( \varvec{\mathcal {I}} + \frac{\partial {\mathbf {W}}^{\text {(ex)}}}{\partial \mathbf {z}} \right) ~\mathrm{d}V_{\mathbf {z}}, \end{aligned}$$

where ${\hat{\varOmega }}_{e}$ is the extracellular space component of the unit we are homogenising over. Remembering that $\sigma _{e}$ is assumed to be constant, the equations for the functions $W_{1}^{\text {(ex)}},W_{2}^{\text {(ex)}},W_{3}^{\text {(ex)}}$ satisfy simpler differential equations than the corresponding functions $W_{1}^{\text {(in)}},W_{2}^{\text {(in)}},W_{3}^{\text {(in)}}$ for the intracellular space, defined by Eqs. (35) and (36). In this case, the functions $W_{1}^{\text {(ex)}},W_{2}^{\text {(ex)}},W_{3}^{\text {(ex)}}$, periodic in $\mathbf {z}$, satisfy

$$\begin{aligned} \nabla _{\mathbf {z}}^{2} W_{j}^{\text {(ex)}}&= 0, \qquad \mathbf {z}\in \varOmega _{e}, \\ \nabla _{\mathbf {z}} W_{j}^{\text {(ex)}} \cdot \mathbf {n}&= - n_{j}, \qquad \mathbf {z}\in \varGamma _{m}. \end{aligned}$$

### The Homogenised Phenotypes Model

Before we can write down the bidomain equations, we need to evaluate the integral that appears on the right-hand side of Eqs. (38) and (40) when the repeating unit that we homogenise over comprises two distinct varieties of cardiac cell. We write *v* as the asymptotic expansion given by

42 $$\begin{aligned} v(\mathbf {x},\mathbf {z},t) = V(\mathbf {x},t) + \delta v_{1}(\mathbf {x},\mathbf {z},t) + \delta ^{2} v_{2}(\mathbf {x},\mathbf {z},t) + \cdots , \end{aligned}$$

We first partition the cell membrane into two regions, $\varGamma _{m1}$ and $\varGamma _{m2}$, where $\varGamma _{m1}$ represents the membrane between the first cell type and the extracellular space, and $\varGamma _{m2}$ represents the membrane between the second cell type and the extracellular space. These two cell types have capacitances $C_{m_{1}},C_{m_{2}}$, and ionic currents $I_{\text {ion},1},I_{\text {ion},2}$. We may then write the integral on the right-hand side of Eqs. (38) and (40) as

$$\begin{aligned}&\int _{\varGamma _{m}} {{\mathcal {A}}}_{1} C_{m} \frac{\partial v}{\partial t} + {{\mathcal {B}}}_{1} I_{\text {ion}}~\mathrm{d}S_{\mathbf {z}} \\&\quad =\int _{\varGamma _{m1}} {{\mathcal {A}}}_{1} C_{m_{1}} \frac{\partial v}{\partial t} + {{\mathcal {B}}}_{1} I_{\text {ion},1}(v;{\mathbf {u}}_{1})~\mathrm{d}S_{\mathbf {z}} + \int _{\varGamma _{m2}} {{\mathcal {A}}}_{1}C_{m_{2}} \frac{\partial v}{\partial t} + {{\mathcal {B}}}_{1} I_{\text {ion},2}(v;{\mathbf {u}}_{2})~\mathrm{d}S_{\mathbf {z}}. \end{aligned}$$

The surface area, *S*, of the membrane within the unit that we homogenise over is partitioned in the proportions $\rho _{1},\rho _{2}$ of the two cell types. Using the asymptotic expansion of *v* given by Eq. (42), we obtain, to leading order,

43 $$\begin{aligned} \int _{\varGamma _{m}} {{\mathcal {A}}}_{1} C_{m} \frac{\partial v}{\partial t} + {{\mathcal {B}}}_{1} I_{\text {ion}}~\mathrm{d}S_{\mathbf {z}}= & {} S \big ( \rho _{1} C_{m_{1}} + \rho _{2} C_{m_{2}} \big ) \frac{\partial V}{\partial t} \nonumber \\&+ S \big ( \rho _{1} I_{\text {ion},1}(V;{\mathbf {u}}_{1}) + \rho _{2} I_{\text {ion},2}(V;{\mathbf {u}}_{2}) \big ). \end{aligned}$$

Using Eqs. (38), (40) and (43), we may write down the bidomain equations for our homogenised system,

44 $$\begin{aligned} \nabla \cdot \left( \varSigma _{i} \nabla \phi _{0}^{\text {(in)}} \right)= & {} \chi \bigg ({{\mathcal {A}}}_{1} C_{m} \frac{\partial V}{\partial t} + {{\mathcal {B}}}_{1} I_{\text {ion}}\bigg ), \end{aligned}$$

45 $$\begin{aligned} \nabla \cdot \left( \varSigma _{e} \nabla \phi _{0}^{\text {(ex)}} \right)&= -&\chi \bigg ({{\mathcal {A}}}_{1} C_{m} \frac{\partial V}{\partial t} + {{\mathcal {B}}}_{1} I_{\text {ion}}\bigg ), \end{aligned}$$

where the homogenised forms of $C_m$ and $I_\text {ion}$ are defined by

46 $$\begin{aligned} C_{m}= & {} \rho _{1}\, C_{m1} + \rho _{2}\, C_{m2}, \end{aligned}$$

47 $$\begin{aligned} I_{\text {ion}}= & {} \rho _{1}\, I_{\text {ion},1}(V;{\mathbf {u}}_{1}) + \rho _{2}\, I_{\text {ion},2}(V;{\mathbf {u}}_{2}), \end{aligned}$$

so that both the homogenised capacitance and ionic current are the proportional contributions from the two cell types, and we have $\chi = S/|\varOmega |$. Note that extending this approach to further cell types would be a simple extension of Eqs. (46) and (47) to add further terms and proportions of each phenotype.

When using the bidomain equations in practice, it is common to eliminate $\phi _{0}^{\text {(in)}}$ from Eqs. (44) and (45), giving

$$\begin{aligned} \chi \bigg ({{\mathcal {A}}}_{1} C_{m} \frac{\partial V}{\partial t} + {{\mathcal {B}}}_{1} I_{\text {ion}}\bigg )= & {} \nabla \cdot \left( \varSigma _{i} \nabla (V + \phi _{0}^{\text {(ex)}}) \right) , \\ 0= & {} \nabla \cdot \left( \varSigma _{i} \nabla V + \left( \varSigma _{i} + \varSigma _{e} \right) \nabla \phi _{0}^{\text {(ex)}} \right) . \end{aligned}$$

### The Partitioned Phenotypes Model

When using the partitioned phenotypes model, we are able to partition the tissue into one set of regions containing only the first phenotype, and another set of regions where only the second phenotype exists. In the first of these regions, we have $\rho _1=1, \rho _2=0$ allowing us to deduce that $C_m=C_{m1}$ from Eq. (46) and that $I_{\text {ion}}=I_{\text {ion},1}$ from Eq. (47). Similarly, in the second region we have $\rho _1=0, \rho _2=1$ and so $C_m=C_{m2}$ and $I_{\text {ion}}=I_{\text {ion},2}$. The solutions to the bidomain equations in these regions are then coupled by demanding that $\phi _{0}^{\text {(in)}}$, $\phi _{0}^{\text {(ex)}}$ and the homogenised intracellular and extracellular currents are continuous across the partition between these regions, i.e.:

$$\begin{aligned} \left( \varSigma _{i} \nabla \phi _{0}^{\text {(in)}} \right) \cdot {\mathbf {n}}, \qquad \text {and} \qquad \left( \varSigma _{e} \nabla \phi _{0}^{\text {(ex)}} \right) \cdot {\mathbf {n}}, \end{aligned}$$

are continuous across the partition boundary, where ${\mathbf {n}}$ is a normal vector to the partition.
